# TRPC5: a new entry to the chromaffin cell’s palette of ion channels that control adrenal response to hypoglycemia

**DOI:** 10.1038/s44318-024-00286-z

**Published:** 2024-11-01

**Authors:** Emilio Carbone

**Affiliations:** Department of Drug Science, Lab of Cell Physiology and Molecular Neuroscience, University of Torino, Torino, Italy

**Keywords:** Genetics, Gene Therapy & Genetic Disease, Metabolism, Neuroscience

## Abstract

A new study implicates TRPC5 channels in adrenaline secretion during hypoglycemia.

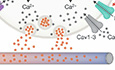

## Teaser

Regulation of glucose levels during insulin-evoked hypoglycemia is impaired in patients with diabetes and can lead to a condition called hypoglycemia-associated autonomic failure (HAAF). The underlying mechanism of the reduced sympathoadrenal response in HAAF patients to counteract hypoglycemia is not yet clarified. In this issue of *The EMBO Journal*, Bröker-Lai et al, show that mice lacking TRPC5 channels possess an impaired response to insulin-induced hypoglycemia similar to humans with HAAF. The altered response is due to a reduced release of adrenaline from chromaffin cells lacking functional TRPC5 channels activated by ACh and PACAP, the neurotransmitters released during sympathetic stimulation. Their work highlights a novel cell signaling pathway in which stimulation of PAC1 and muscarinic M1 receptors activate TRPC5 channels to induce sustained adrenaline secretion during the adrenergic counter-response to hypoglycemia.

Hypoglycemia is an extremely common side effect of insulin therapy experienced mainly by patients with type 1 diabetes mellitus (T1DM). Repeated hypoglycemic episodes that occur in T1DM patients reduce the physiological counter-response to successive hypoglycemic episodes, leading to a condition called hypoglycemia-associated autonomic failure (HAAF), which can result in loss of consciousness, coma, and even death (Senthilkumaran et al, 2016). HAAF is characterized by a reduced sympathoadrenal response to an ongoing hypoglycemic episode but the mechanisms that cause the decreased response to hypoglycemia are still far from being clarified. Further, there are no specific diagnostic tests currently available that predict, or therapies that prevent HAAF.

Although, in principle, a decreased level of insulin or an increased amount of glucagon and other hormones would be sufficient to counter-regulate a hypoglycemic event, it is mainly through adrenaline-driven mechanisms that normal glucose levels are rapidly restored during hypoglycemia in patients with diabetes. While physiologically, circulating adrenaline increases quickly following a hypoglycemic event, in HAAF patients the sympathetically stimulated blood level of adrenaline is dampened. How this occurs is still largely unknown, likely contributing to the lack of therapies for HAAF. Solving this requires an understanding of the hypoglycemia-sensing mechanisms and how a suddenly low glycemia initiates the rapid adrenergic counter-regulatory response.

**Figure 1:**
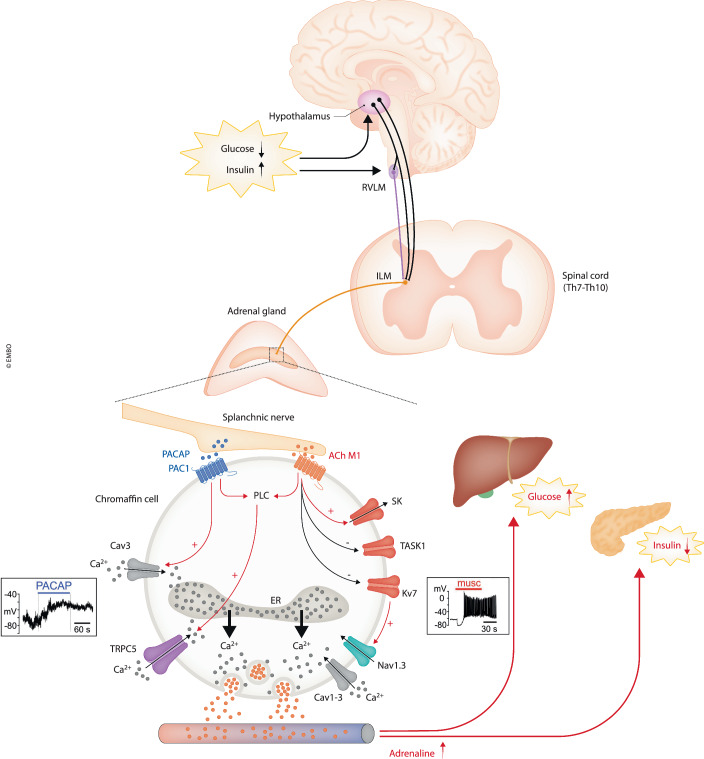
Schematic representation of the TRPC5-mediated adrenergic counter-regulatory response to insulin-induced hypoglycemia. In hypoglycemic conditions (*low glucose*, *high insulin*), the hypothalamic glucose-sensing neurons project directly to the 7–10 thoracic vertebra of the spinal cord (black) and to the glucose-sensitive hindbrain rostral ventrolateral medulla (RVLM) neurons (magenta). Both efferent neurons, from hypothalamus and RVLM, stimulate the sympathetic preganglionic neurons (splanchnic nerves, orange) in the intermediolateral nucleus (IML). The stimulated splanchnic nerves induce the release of PACAP and ACh in a frequency-dependent manner at the adrenergic chromaffin cells of adrenal medulla (dotted square). Activation of PAC1 and muscarinic (M1) receptors induces the activation of phospholipase C and the opening or closing of several ion channels, as illustrated to the bottom. The PAC1-mediated signal proceeds through the activation of TRPC5 (Bröker-Lai et al, 2024) and Cav3 (T-type) channels (Smith and Eiden, 2012) causing long-lasting sub-threshold depolarizations, no sustained APs (rectangle to the left) and marked Ca^2+^-dependent release of adrenaline (red dots). The M1-mediated signal, in parallel to the opening of TRPC5 channels, opens SK channels (+, red) and closes TASK1 and Kv7 potassium channels (−, black), thus causing above-threshold depolarization that opens Nav1.3 and Cav1-3 channels (+, red arrow), generates sustained AP trains (rectangle to the right) and initiates a Ca^2+^-dependent release of adrenaline (Carbone et al, 2019). Cytoplasmic Ca^2+^ (black dots) is raised by Ca^2+^-entry through TRPC5, Cav channels and Ca^2+^ released from the endoplasmic reticulum (ER) through the Ca^2+^-induced Ca^2+^ release indicated by the black arrows (Tuluc and Carbone, 2024). The increased adrenaline in blood plasma increases glucose in the blood by stimulating gluconeogenesis and glycogenolysis in liver hepatocytes and inhibiting insulin production in pancreatic β-cells.

Many brain areas, including hypothalamic regions and the hindbrain, are known to regulate blood glucose homeostasis (Verberne et al, 2014). Among them, a number of specialized hypothalamic neurons and catecholaminergic neurons in the hindbrain rostral ventrolateral medulla (RVLM) project directly to the sympathetic preganglionic neurons located in the intermediolateral nucleus (IML) in the spinal cord named splanchnic nerves (Fig. 1, top). Interestingly, the RVLM hindbrain area contains neurons that generate stress-induced hyperglycemia, which drives the “fight-or-flight” response fundamental for survival.

As a final step, the sympathetic splanchnic nerve in the IML projects to the chromaffin cells (CCs) of the adrenal medulla and stimulate the release of adrenaline via acetylcholine (ACh) and pituitary adenylate cyclase-activating polypeptide (PACAP) in a frequency-dependent manner (Carbone et al, 2019). While the signaling events of catecholamine release mediated by ACh, via nicotinic (nAChR) and muscarinic (mAChR) receptors, have been described in great detail, the molecular pathways downstream of PACAP receptor (PAC1) activation are still largely unknown (Smith and Eiden, 2012). PAC1 receptor activation in CCs causes sub-threshold (ΔV ~ 20 mV) long lasting depolarizations (tens of seconds) that activate low-threshold T-type (Cav3) channels able to sustain catecholamine release (Fig. 1, bottom-left). PACAP also likely activates a 2-APB-sensitive cation channel, with inward current characteristics similar to those carried by TRPC channels.

Promoting Na^+^ and Ca^2+^ influx into the cell, TRPC channels (TRPC1–TRPC7) contribute to membrane depolarization and induce Ca^2+^-dependent intracellular signaling in neurons and neuroendocrine cells. In bovine and mouse CCs, TRPC1 channels are shown to contribute to catecholamine release when CCs are stimulated by bradykinin or histamine through a PLCβ-mediated pathway (Marom et al, 2011). Ca^2+^-entry through TRPC5 channels, is shown to regulate the firing of insulin-, leptin-, and serotonin-evoked excitations of hypothalamic anorexigenic Pomc neurons, thereby part of a central mechanism that regulates glucose metabolism (Gao et al, 2017). Thus, it seems likely that TRPC channels may act as central regulators of the autonomic counter-response to hypoglycemia either at the start (hypothalamus) or at the endpoint (chromaffin cells) of the hypothalamic-sympatho-adrenal (HSA) axis.

In this issue of EMBO J, using a combination of TRPC-deficient mouse models (Trpc1/4/5/6^−/−^ and Trpc5^−/−^) and ad-hoc pharmacological tools, Bröker-Lai et al, bring overwhelming evidence that TRPC5 is highly expressed in CCs and its functional loss leads to a defective counter-regulation to insulin-evoked hypoglycemia due to the lack of increased plasma adrenaline (Bröker-Lai et al, 2024). To reach these conclusions the authors undertook a series of preliminary experiments to create and test a population of WT and mutated diabetic animals using either the insulin or glucose tolerance test and showed that *Trpc1/4/5/6*^*–/–*^ and *Trpc5*^*–/–*^ mice develop an aggravated insulin-induced hypoglycemia associated with reduced plasma adrenaline. After having established the *Trpc5*^*–/–*^ mouse phenotype, the authors show that supplementation of adrenaline or Englerin A (EA), an activator of TRPC4/TRPC5 channels, can mitigate the aggravated hypoglycemia conditions in TRPC5-deficient mice. Given this, and after having shown that TRPC5 is critical to the elevated adrenaline during insulin-induced hypoglycemia, the authors tested whether genetic loss of TRPC5 in RVLM neurons is critical to the autonomous counter regulation maintenance and found that is not. They finally showed that TRPC5 activity loss at the endpoint of the HSA axis, in chromaffin cells, is responsible for the impaired PACAP- and muscarine-evoked catecholamine secretion.

Following this, Bröker-Lai et al, focused on the role of TRPC5 channels in the regulation of adrenaline release from adrenal CCs. The authors nicely demonstrate that TRPC5 is the final target of the PACAP-mediated regulation of adrenaline release from mouse CCs. Application of either 1 µM PACAP or 30 nM EA produced a marked increase of cytoplasmic Ca^2+^ as well as sustained catecholamine release, visualized by an increased frequency of amperometric quantal secretory events that was strongly attenuated in TRPC5-deficient mice. In agreement with previous reports (Smith and Eiden, 2012), Bröker-Lai et al, also show that the selective agonist PACAP-38 causes slowly-developing depolarizations of small amplitude, insufficient to activate voltage-gated Na^+^ and Ca^2+^ channels, thus unable to produce repetitive action potential (AP) firings that are typical of mouse CCs (see Fig. 1, bottom-left). The depolarization and relative inward current produced by TRPC5 activation are absent in TRPC5-deficient mouse CCs. Thus, TRPC5 appears as a new unexpected entry in the rich palette of functional ion channels expressed by CCs and actively involved in the control of catecholamine secretion during sympathetic stimulation (Lingle et al, 2018).

Of relevance is also the observation that the muscarine-mediated increase of cytoplasmic Ca^2+^ and catecholamine secretion is also strongly attenuated in TRPC5-deficient mouse CCs, demonstrating that the two neurotransmitters (ACh and PACAP) simultaneously released during stressful stimulation both act on TRPC5 channels (Fig. 1, bottom). This finding is of key importance and warrants some consideration. As detailed in (Carbone et al, 2019), muscarine acts on CCs by transiently activating SK channels and steadily inhibiting TASK and Kv7 channels, thus producing sustained depolarization and AP firing (Fig. 1, bottom-right). Blocking K^+^ channels rapidly opens voltage-gated Nav1.3 and Cav1-3 (L, N, P/Q and T-type) channels which starts the Ca^2+^-dependent release of adrenaline from CCs. This AP-dependent Ca^2+^-entry driven by Cav channels goes in parallel with the AP-independent Ca^2+^-entry through open TRPC5 channels. How do the two Ca^2+^ influxes contribute to the overall Ca^2+^ signal that drives catecholamine release and, more importantly, why does the loss of TRPC5 channels nearly nullify the release of catecholamines (Fig. 5P in Bröker-Lai et al (2024))? The question is intriguing and will most likely be solved by considering that, during sustained stimulation, the major Ca^2+^ supply for catecholamine secretion in CCs comes from the Ca^2+^-induced Ca^2+^-release mediated by the RyR and IP_3_ receptors of the endoplasmic reticulum as recently demonstrated by Antonio G. García (Madrid) and Arturo Hernández-Cruz labs (Mexico City) and discussed in a short review by (Tuluc and Carbone, 2024).

In conclusion, the work by Bröker-Lai et al, conveys conclusive evidence about the critical role that TRPC5 plays in the generation of PACAP-mediated release of catecholamines from CCs during stressful stimulation but also opens to new future studies concerning the “common” or “distinct” role that muscarine and PACAP have in the control of catecholamines release from CCs during stressful sympathetic stimulation. The finding that PACAP and muscarine both act on the same TRPC5 channel of CCs should suggest some caution in concluding that PACAP acts as “master regulator” of stress signaling (Eiden et al, 2018). The effective role of M1 muscarinic receptors in the long-lasting control of catecholamine release from CCs should be taken into proper consideration.

From a clinical perspective, the work by Bröker-Lai et al, has the great merit of uncovering a new role for TRPC5 in the control of the adrenergic counter-response to hypoglycemia and suggests TRPC5 as a likely potential target for diagnostic and pharmacological intervention in patients with diabetes and HAAF complications.
